# Supplementary material for: Data-driven decentralized breeding increases prediction accuracy in a challenging crop production environment
Source: Commun Biol. 2021 Aug 19;4:944. doi: 10.1038/s42003-021-02463-w (PMC8376984; doi:10.1038/s42003-021-02463-w)
Supplement: Supplementary file 2 — Supplementary Information [file 42003_2021_2463_MOESM2_ESM.pdf]

Supplementary Materials for

Data-driven decentralized breeding increases prediction accuracy in a challenging crop production environment

Kauê de Sousa, Jacob van Etten, Jesse Poland, Carlo Fadda, Jean-Luc Jannink, Yosef Gebrehawaryat Kidane, Basazen Fantahun Lakew, Dejene Kassahun Mengistu, Mario Enrico Pè, Svein Øivind Solberg, Matteo Dell'Acqua
Correspondence to: m.dellacqua@santannapisa.it

This PDF file includes:
Supplementary Note
Supplementary Figures 1 to 16

Other Supplementary Materials for this manuscript include the following:

Supplementary Data 1: Broad sense heritability (H^2) and narrow sense heritability (h^2) of phenotypic traits measured in centralized stations. For each trait, the table reports heritability values for different location and year combinations. DB, days to booting; DF, days to flowering; DM, days to maturity; PH, plant height; NET, number of effective tillers; SPL, spike length; SPS, seeds per spike; BM, biomass; GY, grain yield; TGW, thousands grain weight.

Supplementary Data 2: Broad sense heritability (H^2) and narrow sense heritability (h^2) of overall appreciation (OA) provided by farmers in centralized stations. The table reports heritability values for different location and gender combinations.

Supplementary Data 3: Prediction accuracy of the benchmark for GY and OA in different prediction scenarios. For each scenario, the table report a brief description. Training set and validation set are reported with the corresponding number of genotypes in brackets. For both training and validation, total number of data points is derived from number of genotypes, replicas, years, locations. Accuracy and standard deviation of each scenario are provided.

Supplementary Data 4: Mean and standard deviation of the prediction accuracy (Kendall τ) of 3D-breeding in 100 simulations run for each of five reduced scenarios.

Supplementary Note

Derivation of best linear unbiased prediction (BLUP) values

$GY_{STATION}$ and $OA_{STATION}$ measured in centralized trials were used to derive best linear unbiased prediction (BLUP) values using the R package ASReml[1]. BLUPs for $GY_{STATION}$ were derived with the following model:

Equation [s1]

$$y_{ijkn} = \mu + g_i + y_j + l_k + gl_{ij} + yl_{jk} + b_{n(jk)} + e$$

In Equation s1, y_{ijkn} is the observed phenotypic value, μ is the overall mean, g_i is the random effect of genotype for entry i, y_j is the random effect for year j, l_k is the fixed effect for location k, gl_{ik} is the random interaction effect between genotype i and location k, yl_{jk} is the random interaction between year j and location k, b is the random effect of replicated block nested within year j and location k, and e is the error. The model considers locations a fixed factor included by experimental design. The year effect is considered random because it cannot be controlled experimentally.

For calculation of BLUPs with a single location, the data was sub-set by location and Equation [s1] was reduced as follows:

Equation [s1.1]

$$y_{ijn} = \mu + g_i + y_j + b_{n(j)} + e$$

Where, y_{ijn} is the observed phenotypic value, μ is the overall mean, g_i is the random effect of genotype for entry i, y_j is the random effect for year j, b is the random effect of replicated block nested within year j and e is the error. This model was calculated independently for each location.

Likewise, for calculation of BLUPs with a single year, the data was subset by year and Equation [s1] was reduced to:

Equation [s1.2]

$$y_{ikn} = \mu + g_i + l_k + gl_{ik} + b_{n(k)} + e$$

Where, y_{ikn} is the observed phenotypic value, μ is the overall mean, g_i is the random effect of genotype for entry i, l_k is the fixed effect for location k, gl_{ik} is the random interaction between genotype i and location k, b is the random effect of replicated block nested within location k, and e is the error.

Broad-sense heritability (H^2) of measured traits was derived from the variance component estimates from Equation [s1] as follows:

Equation [s2]

$$H^2 = \frac{\sigma_g}{(\sigma_g + \frac{\sigma_{gl}}{n_{loc}} + \frac{\sigma_{gy}}{n_{year}} + \frac{\sigma_e}{n_{rep} * n_{loc} * n_{year}})}$$

Where σ_g is the genetic variance, σ_{gl} is the genotype by location variance, and σ_e is the error variance, n_{loc} is the number of locations, n_{year} is the number of years, and n_{rep} is the number of replications.

For calculation of heritability within a location, variance components were estimated from Equation [s1.1] and broad-sense heritability was calculated as follows:

Equation [s2.1]

$$H^2 = \frac{\sigma_g}{(\sigma_g + \frac{\sigma_{gy}}{n_{year}} + \frac{\sigma_e}{n_{rep} * n_{year}})}$$

For calculation of heritability within a single year, variance components were estimated from Equation [s2.2] and broad-sense heritability calculated as:

Equation [s2.2]

$$H^2 = \frac{\sigma_g}{(\sigma_g + \frac{\sigma_{gl}}{n_{loc}} + \frac{\sigma_e}{n_{rep} * n_{loc}})}$$

With each component defined as in Equation [s2].

Finally, for estimation of broad-sense heritability within a location – year the following equation was used:

Equation [s2.3]

$$H^2 = \frac{\sigma_g}{(\sigma_g + \frac{\sigma_e}{n_{rep}})}$$

Narrow sense heritability (h^2) of measured traits was obtained linking the inverse genetic relationship matrix derived from the R package AGHmatrix[2] to regressor variables in Equation [s1] and using the deriving variance components in Equation [s2] and derived Equation [s2.1], Equation [s2.2], and Equation [s2.3].

The general model to derive BLUPs of $OA_{STATION}$ was similar to the equations used for metric traits with the addition of variance estimates for gender, farmer groups and individual farmers. For analysis of this data we considered gender similar to a two-factor treatment with farmer groups as a blocking effect and individual farmers as repeated measures. This provides a nested blocking design. As the farmer scores were only recorded in one year, the model terms are consistent with the evaluation of metric traits in multiple locations within a single year (Equation [s1.2]).

Equation [s3]

$$y_{ikmnqtxy} = \mu + g_i + l_k + p_m + gl_{ik} + pl_{mk} + b_{n(k)} + d_{q(mk)} + gpl_{imk} + gd_{iq(mk)} + f_{t(qmk)} + r_{x(nk)} + c_{y(nk)} + grcl_{ixyk} + e$$

In Equation s3, y_{ijk} is the observed trait value, μ is the overall mean, g_i, l_k, gl_{ik}, and $r(l)_{nk}$ are consistent with Equation [2], p_m is the random effect for gender m, pl_{mk} is the interaction between gender and location, gp_{im} is the genotypic interaction with gender, $d_{(q(mk))}$ is the random effect of group q nested within gender and location, gpl_{imk} is the interaction of genotype with gender and location, $gd_{(iq(mk))}$ is the interaction of genotype by group within gender and location, $r_{(x(nk))}$ and $c_{(y(nk))}$ are the random effect of row x and column y nested within replication and location. Finally, we model the within plot variance considering farmers as repeated measures where $f_{(t(qmk))}$ is the random effect of farmer nested within group, gender and location and the within plot variance is captured in the term $grcl_{ixyk}$ which is the random interaction of genotype by row and column within location (e.g. a single plot). Finally, e is the residual model error assumed to be random and normally distributed.

As with the metric traits, the above model was reduced to estimate variance components to calculate BLUPs within each location, using subsets of the data by location as follows:

Equation [s3.1]

$$y_{imnqtxy} = \mu + g_i + p_m + d_{q(mk)} + gp_{im} + gd_{iq(m)} + f_{t(qm)} + r_{x(n)} + c_{y(n)} + grcl_{ixy} + e$$

Equation [s3.1] is a reduced form of Equation [s3] where, $y_{imnqtxy}$ is the observed trait value, μ is the overall mean, g_i is the random genotypic effect, p_m is the random effect for gender m, gp_{im} is the genotypic interaction

with gender, $d_{(q(m))}$ is the random effect of group q nested within gender, $gd_{(iq(m))}$ is the interaction of genotype by group within gender, $r_{(x(n))}$ and $c_{(x(n))}$ are the random effect of row x and column y nested within replication. As previously, the within plot variance considering farmers as repeated measures is modeled where $f_{(t(qm))}$ is the random effect of farmer nested within group and gender. The within plot variance is designated grc_{ixy} which is the random interaction of genotype by row and column (e.g. a single plot). Finally, e is the residual model error assumed to be random and normally distributed.

To calculate BLUPs and estimate variance components for each gender, the data was subset by gender and Equation [s3] was reduced to:

Equation [s3.2]

$$y_{iknqtxy} = \mu + g_i + l_k + gl_{ik} b_{n(k)} + d_{q(mk)} + gd_{iq(k)} + f_{t(qk)} + r_{x(nk)} + c_{y(nk)} + grcl_{ixyk} + e$$

Each of these terms are consistent with Equation [s3], with specification that $d_{(q(k))}$ is the random effect of group q nested within location, $gd_{(iq(k))}$ is the interaction of genotype by group within location, and $f_{(t(qk))}$ is the random effect of farmer nested within group and location. The within plot variance is again captured in the term $grcl_{ixyk}$ which is the random interaction of genotype by row and column within location (e.g. a single plot). Finally, e is the residual model error assumed to be random and normally distributed.

Finally, we calculated BLUPs for each gender within each location, subsetting the data by both gender and location, using the following model:

Equation [s3.3]

$$y_{inqtxy} = \mu + g_i + b_{n(k)} + d_q + gd_{iq} + f_{t(q)} + r_{x(n)} + c_{y(n)} + grcl_{ixy} + e$$

Each of these terms are consistent with Equation [s3] and Equation [s3.2], with specification that d_q is the random effect of group q, gd_{iq} is the interaction of genotype by group, and $f_{(t(q))}$ is the random effect of farmer nested within group. The within plot variance is captured in the term grc_{ixy} which is the random interaction of genotype by row and column (e.g. a single plot). Finally, e is the residual model error assumed to be random and normally distributed.

The H^2 of OA was derived according to a repeated measures design from the following equation: Equation [s4]

$$H^2 = \frac{\sigma_g}{(\sigma_g + \frac{\sigma_{gd}}{n_{gender}} + \frac{\sigma_{gl}}{n_{loc}} + \frac{\sigma_{gdl}}{n_{gender} * n_{loc}} + \frac{\sigma_w}{n_{rep} * n_{loc})} + \frac{\sigma_w}{n_{rep} * n_{loc} * n_{farmer}})}$$

In Equation [s4], σ_g is the genetic variance, σ_{gd} is the variance of genotype by gender interaction divided by two (for genders), σ_{gl} is the genotype by location variance, σ_{gdl} is the genotype by gender by location variance, σ_w is the within plot genetic variance from sub-sampling specified in Equation [3] as $grcl_{ixyk}$ and σ_e is the error variance. The variance components are respectively divided by the number of locations ($n_{loc} = 2$), the number of replication blocks ($n_{rep} = 2$), and the total number of farmers ($n_{farmer} = 30$).

For the reduced models in Equation [s3.1], Equation [s3.2] and Equation [s3.3] the above heritability calculation was modified as follows.

For the model by location the following calculations were used:

Equation [s4.1]

$$H^2 = \frac{\sigma_g}{(\sigma_g + \frac{\sigma_{gd}}{n_{gender}} + \frac{\sigma_w}{n_{rep}} + \frac{\sigma_e}{n_{rep} * n_{farmer}})}$$

These terms are consistent with Equation [s4]

For the model reduced to within gender estimates the heritability was calculated as follows:

Equation [s4.2]

$$H^2 = \frac{\sigma_g}{(\sigma_g + \frac{\sigma_{gl}}{n_{loc}} + \frac{\sigma_w}{n_{rep} * n_{loc}} + \frac{\sigma_e}{n_{rep} * n_{loc} * n_{farmer}})}$$

In this case the number of farmers is reduced by half (n=15). All terms are likewise consistent with Equation [s4].

Finally, the heritability was estimated for each gender separately within each location as:

Equation [s4.3]

$$H^2 = \frac{\sigma_g}{(\sigma_g + \frac{\sigma_w}{n_{rep}} + \frac{\sigma_e}{n_{rep} * n_{farmer}})}$$

In this case the number of farmers is again half of the total (n=15) and terms are consistent with Equation [s4].

Narrow sense heritability (h^2) of OA was derived linking the inverse genetic relationship matrix derived from the R package AGHmatrix[2] to regressor variables in Equation [s3]. Variance components were used to calculate (h^2) with Equation [s4] and derived Equation [s4.1], Equation [s4.2], and Equation [s4.3].

Influence of data volume on the comparison between 3D-breeding vs centralized breeding

The comparison between the two approaches should be fair. We consider that the two setups are comparable in terms of the costs and efforts but this is difficult to quantify for a situation in which each of these approaches would be used on a routine basis. One key difference, however, is the volume of data available to each, and this is also a key cost driver. Centralized breeding involved 8 plots per genotype (two locations, two years, two replications per location), while decentralized observations were conducted on 113 plots per genotype in the tricot incomplete block design. So, 3D-breeding has many more datapoints per genotype. To assess the sensitivity of 3D-breeding to the data volume for its prediction accuracy, we created 5 scenarios that represent different data volumes available. Each scenario is a subset of the plots containing respectively 75% (85 plots), 50% (56 plots), 25% (28 plots), 15% (17 plots) and 5% (5 plots) of the data. We selected plots randomly, keeping balance between seasons, and always including all 41 genotypes evaluated. For each prediction we calculated the Kendall τ correlation with the observed data. This process was repeated 100 times per scenario and averaged. We report the average Kendall τ correlation for the different scenarios in Table S4.

References

1. Gilmour, A. R., Gogel, B. J., Cullis, B. R., Welham, S. J. & Thompson, R. *ASReml User Guide Release 4.1.* (Structural Specification, VSN International Ltd, Hemel Hempstead, HP1 1ES, 2015).

2. Rampazo Amadeu, R., Cellon, C., Olmestead, J. W., Franco Garcia, A. A. & Resende Jr, M. F. AGHmatrix: R Package to Construct Relationship Matrices for Autotetraploid and Diploid Species: A Blueberry Example. *The Plant Genome* **9**, 1–10 (2016).

Supplementary figures

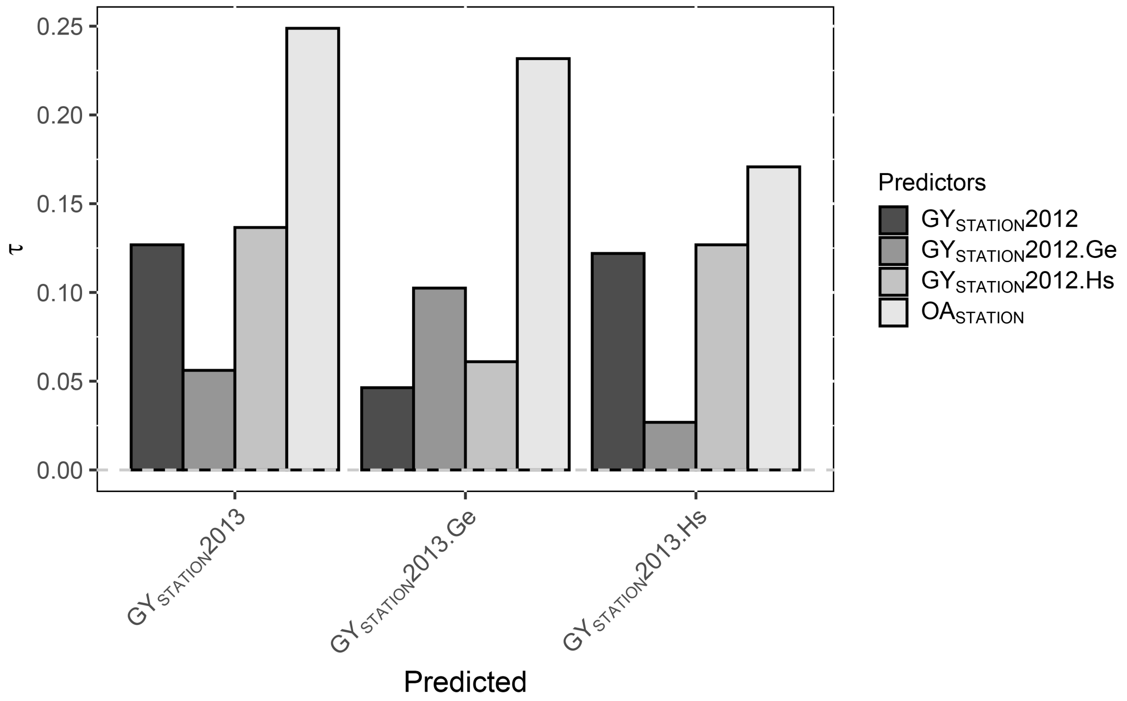

Supplementary Figure 1. Accuracy of genomic prediction within centralized trials, using $GY_{STATION}$ and $OA_{STATION}$ from 2012 to predict traits measured in 2013 data in the same locations (Ge, Geregera; Hs, Hagreselam).

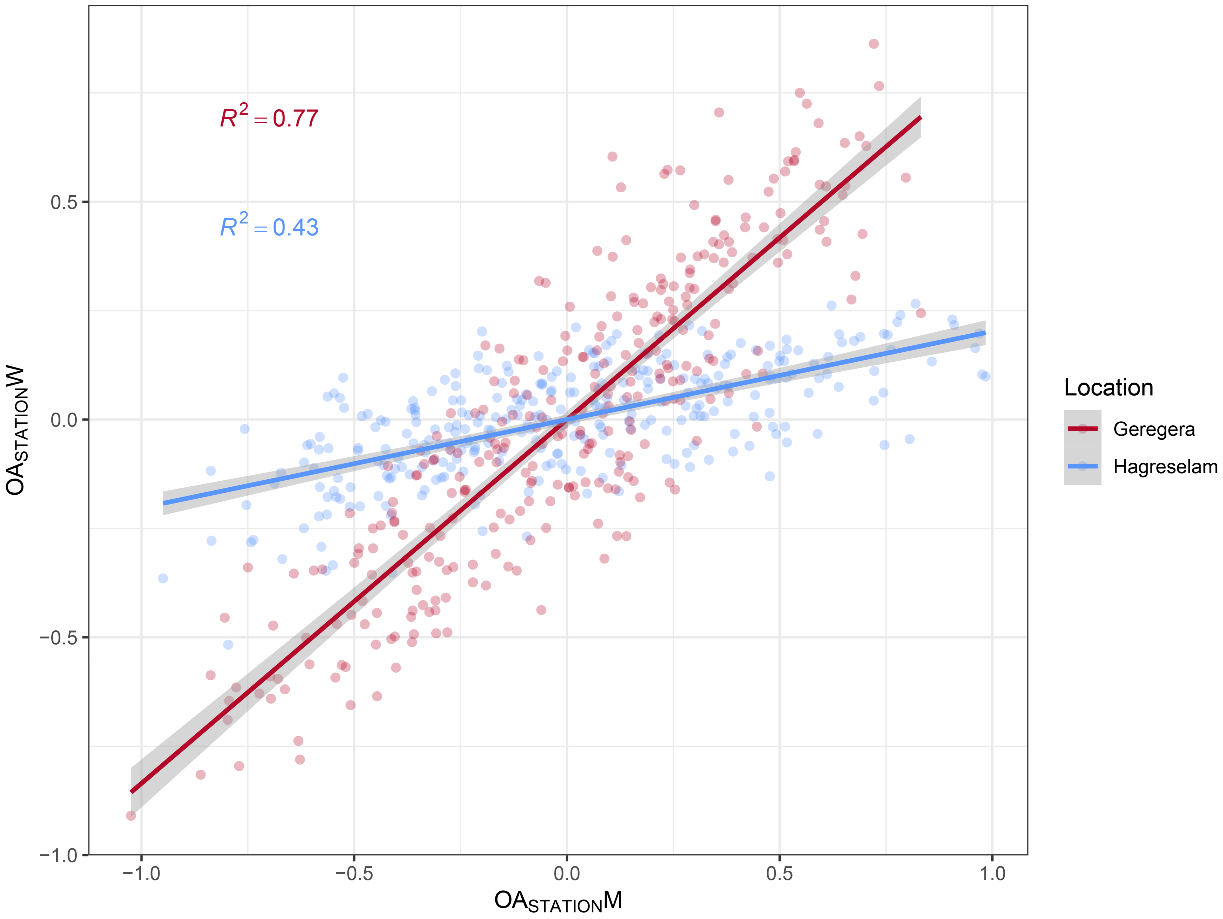

Supplementary Figure 2. Farmer scores agreement across genders in the two centralized stations, Geregera and Hagreselam. On the x-axis, BLUP values for male farmers' OA. On the y-axis, BLUP values for female farmers' OA. A linear model fit is displayed with the corresponding R^2 with colors according to legend.

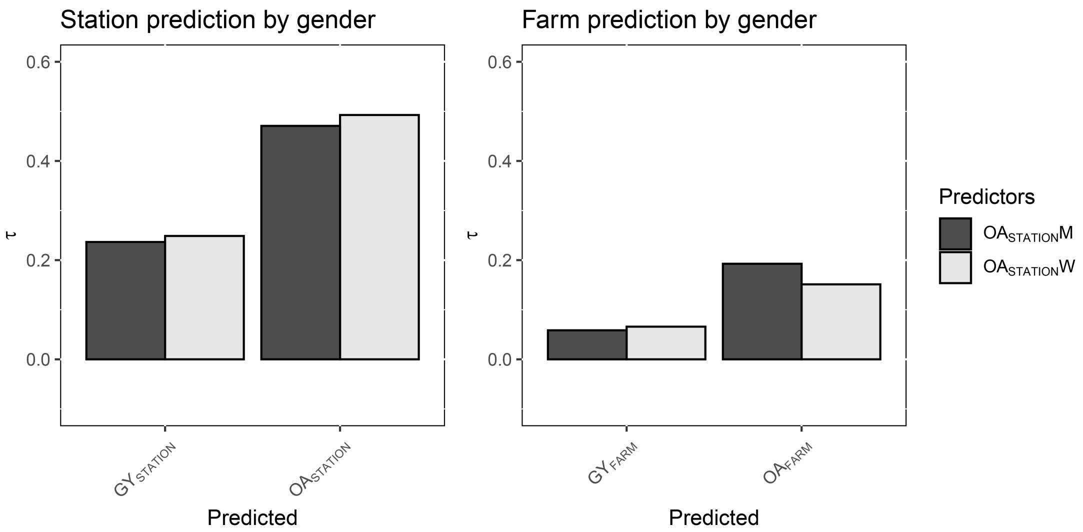

Supplementary Figure 3. Accuracy of genomic prediction by gender (M, men; W, women). In the first panel, gender-specific $OA_{STATION}$ is used to predict $GY_{STATION}$ and $OA_{STATION}$. In the second panel, the same predictors are applied to GY_{FARM} and OA_{FARM}

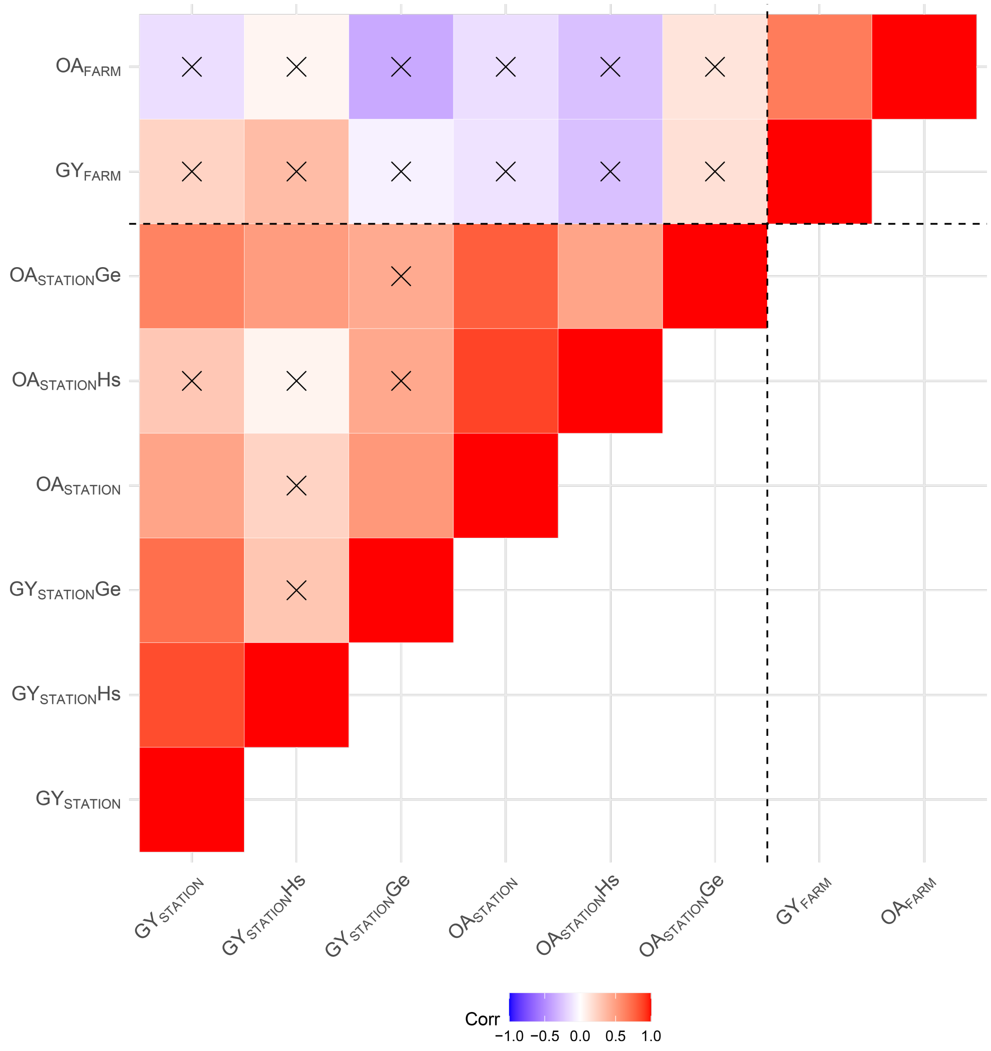

Supplementary Figure 4. Spearman correlations between traits measured in stations and farms. Also given for each location (Ge, Geregera; Hs, Hagreselam) values are derived from log-worth across all decentralized trials. Non-significant correlations are crossed out

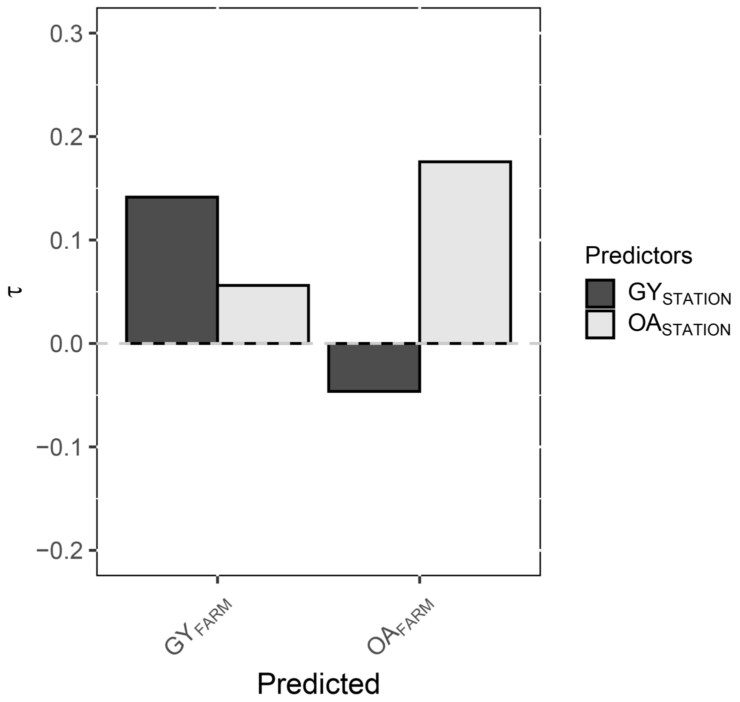

Supplementary Figure 5. Accuracy of genomic prediction using $OA_{STATION}$ and $GY_{STATION}$ to predict GY_{FARM} and OA_{FARM} across seasons.

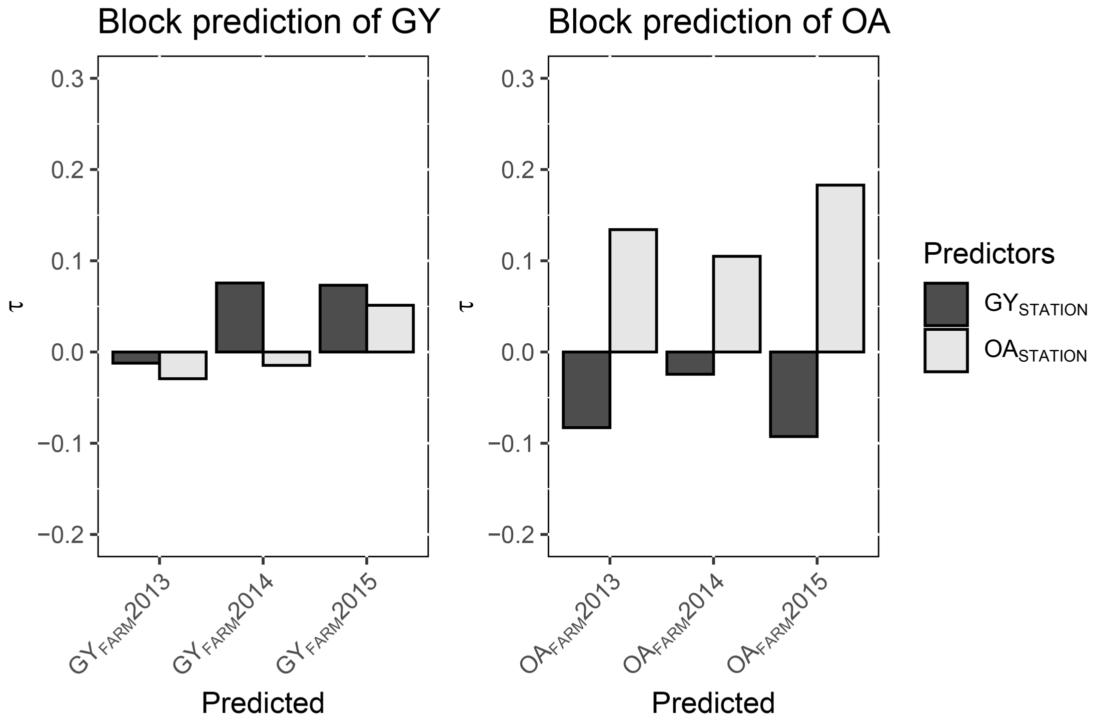

Supplementary Figure 6. Accuracy of genomic prediction using $OA_{STATION}$ and $GY_{STATION}$ to predict GY_{FARM} and OA_{FARM}, restring farm data individual seasons (2013, 2014, 2015)

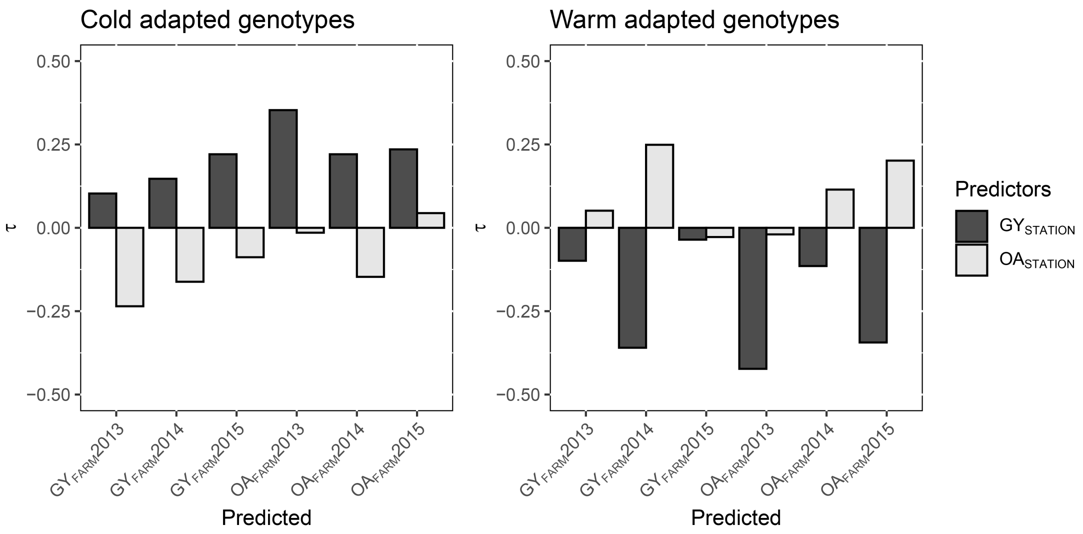

Supplementary Figure 7. Accuracy of genomic prediction using $OA_{STATION}$ and $GY_{STATION}$ to predict GY_{FARM} and OA_{FARM}, training and testing the model on the subset of cold adapted genotypes (left) or warm adapted genotypes (right).

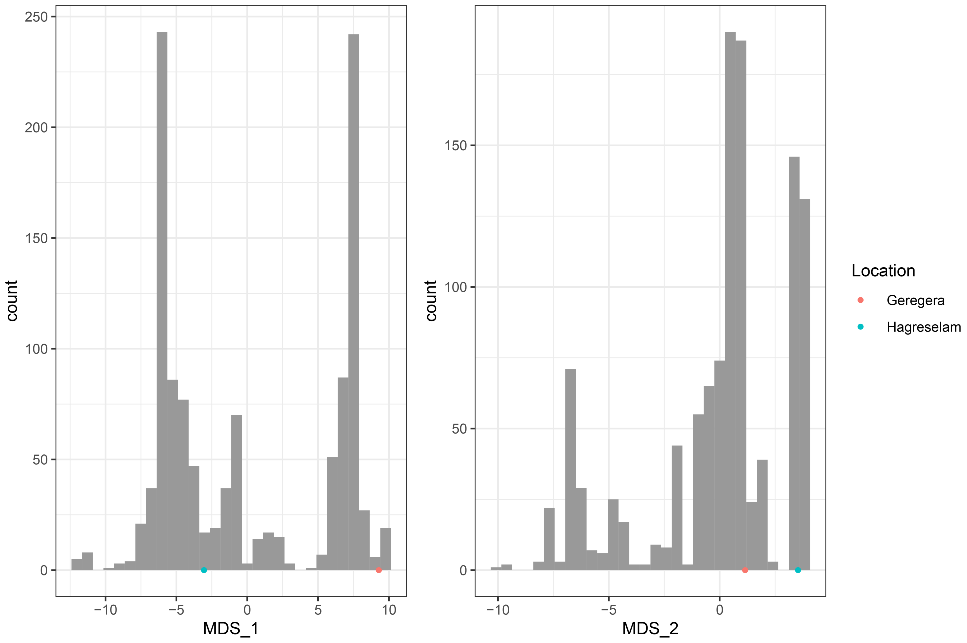

Supplementary Figure 8. Distribution of environmental distance across the test sites, by multidimensional scaling (MDS) of temperature variables. The first MDS dimension is given in the left panel, while the second MDS is given in the panel to the right. The histogram represents the distribution of decentralized fields, while stations are highlighted with dots colored according to legend.

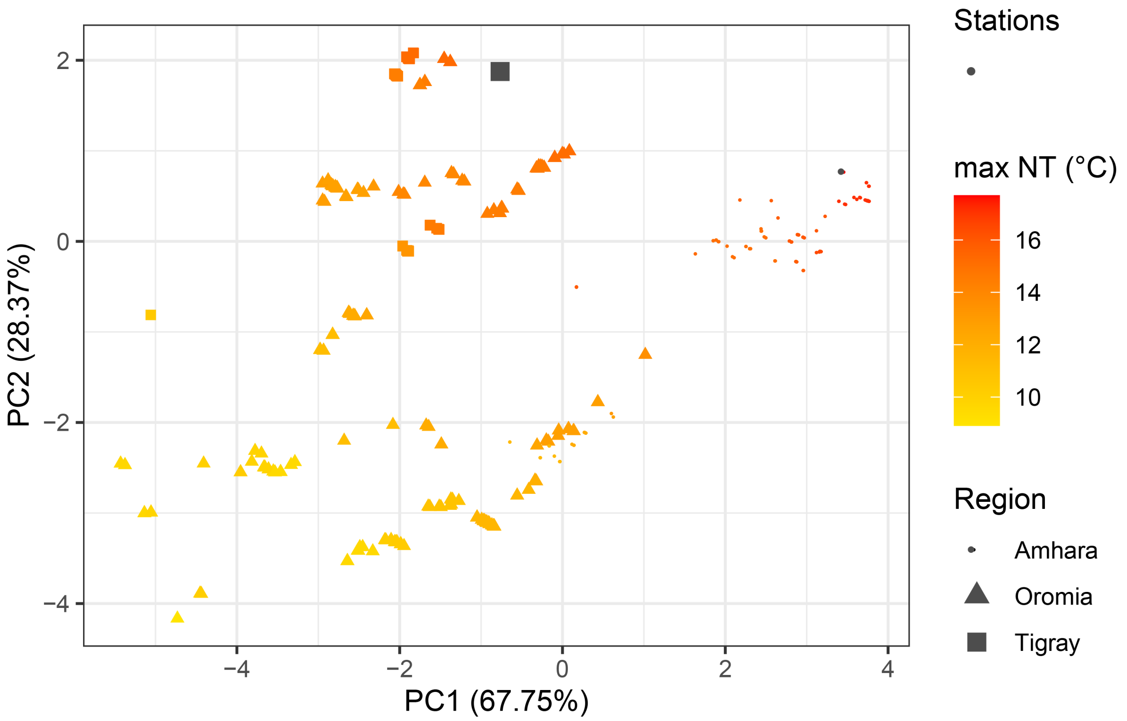

Supplementary Figure 9. A principal components (PC) analysis reporting the environmental diversity at the test sites. PC1 and PC2 are reported with the corresponding proportion of variance explained. Symbols represent test sites with shapes according to administrative regions of Ethiopia. Colored shapes represent farm sites and are colored in increasing shades of red proportionally to the maximum night temperature during reproduction (maxNT) as reported in legend. Stations are represented in bigger size and grey color.

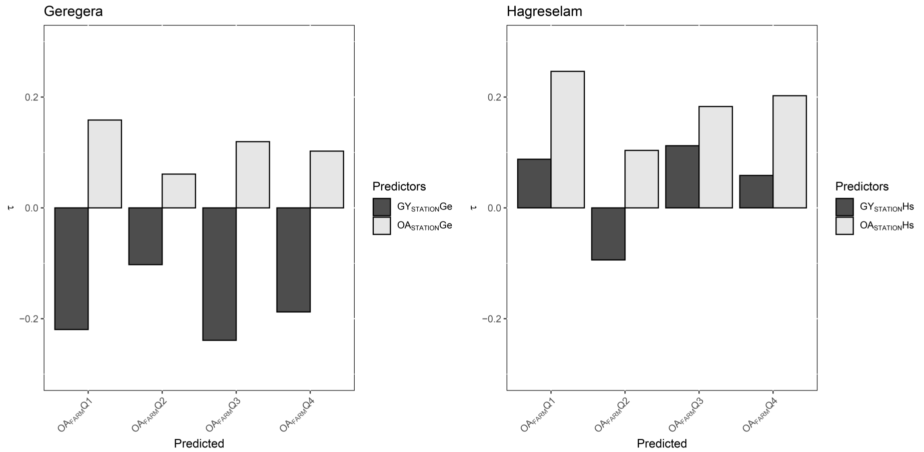

Supplementary Figure 10. Accuracy of genomic prediction using $GY_{STATION}$ and $OA_{STATION}$ to predict GY_{FARM} and OA_{FARM} in increasingly different climatic conditions. GY_{FARM} and OA_{FARM} are computed for farms in the first, second, third, and fourth quantiles (Q1 to Q4) of environmental distances from Geregera (left panel) and Hagreselam (right panel).

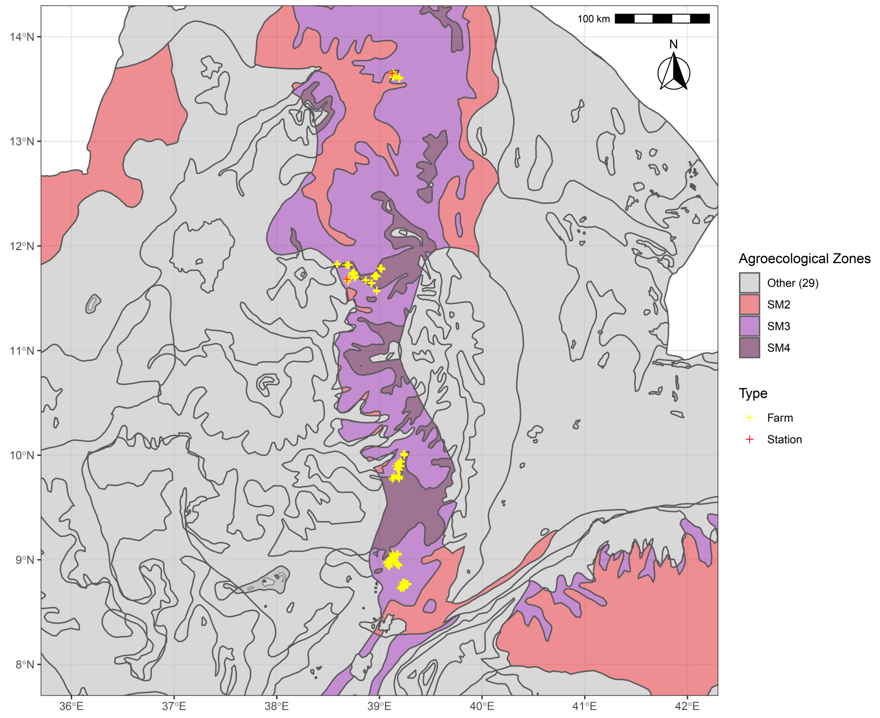

Type	Site	SM2	SM3	SM4
Farm	Amhara	0	153	318
Farm	Oromia	0	332	67
Farm	Tigray	152	143	0
Station	Geregera	0	0	1
Station	Hagreselam	1	0	0

Supplementary Figure 11. A map of Ethiopia reporting the agroecological zones, with colors according to legend. Yellow crosses on the map represent farm sites, red crosses represent stations. A summary is given in the table to the right. Decentralized farms are located in Amhara, Oromia and Tigray and cover warm sub-moist highlands (SM2), tepid sub-moist highlands (SM3), and cool sub-moist highlands (SM4). centralized stations are located in Geregera (Amhara) and Hagreselam (Tigray), and cover SM2 and SM4, though the three agroecologies are in close connection.

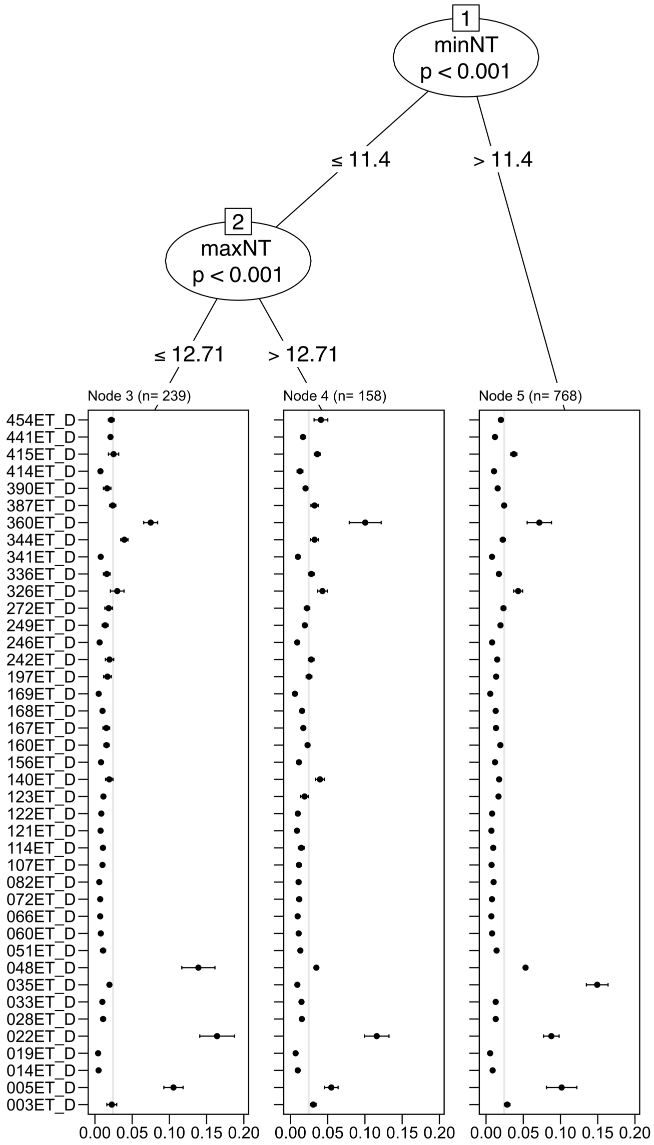

Supplementary Figure 12. Plackett-Luce Tree of decentralized trial data and associated environmental and genomic data for durum wheat in Ethiopia. Intervals show quasi-standard errors. The grey vertical lines indicate the average probability of winning (1 / number of genotypes). In this case, the model selected minNT, the minimum night temperature (Celsius) during the vegetative and maxNT, the maximum night temperature (Celsius) during reproductive period, as the covariate.

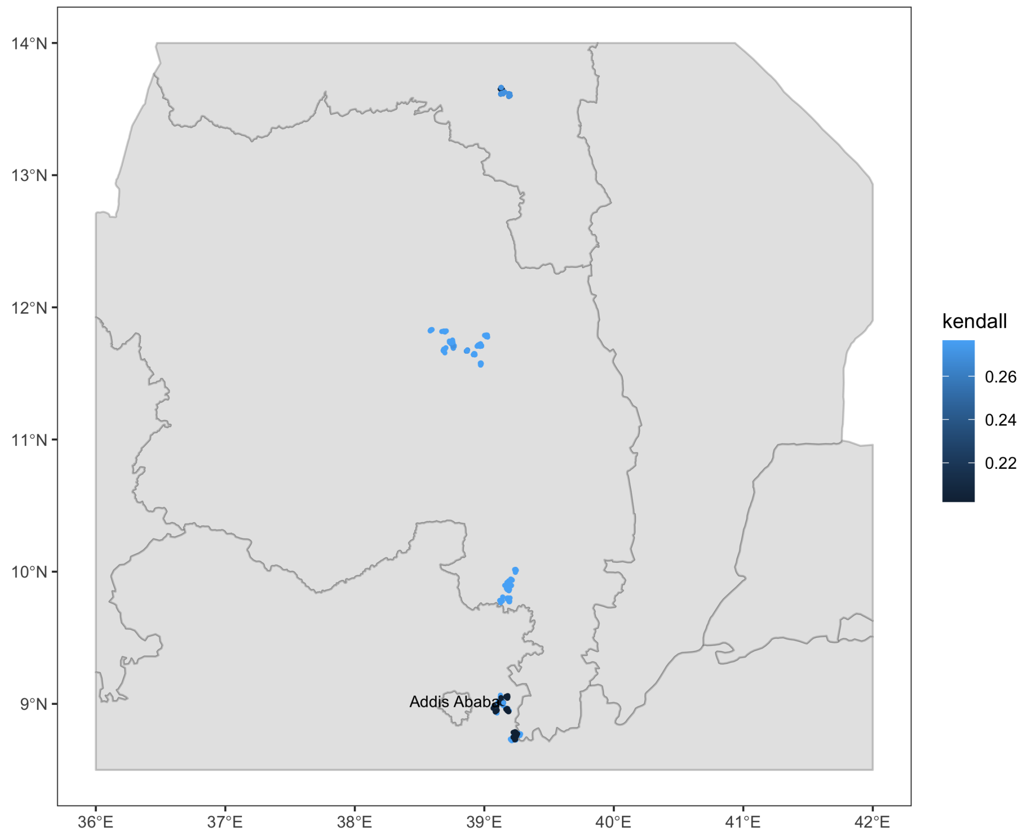

Supplementary Figure 13. Kendall correlation of crop performance on OA_{FARM} across the decentralized plots in Ethiopia

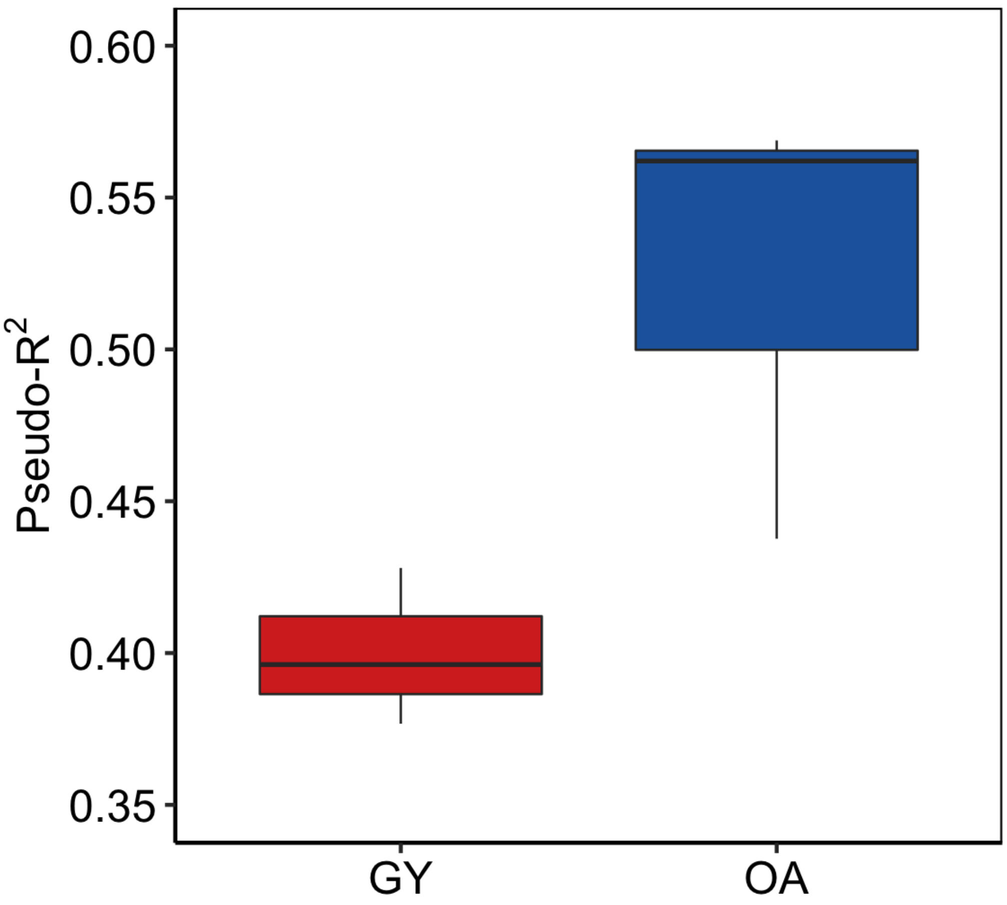

Supplementary Figure 14. Goodness-of-fit (pseudo-R^2) of Plackett-Luce Trees determined with blocked cross-validation for 3D-breeding with grain yield (GY) and farmers' overall appreciation (OA).

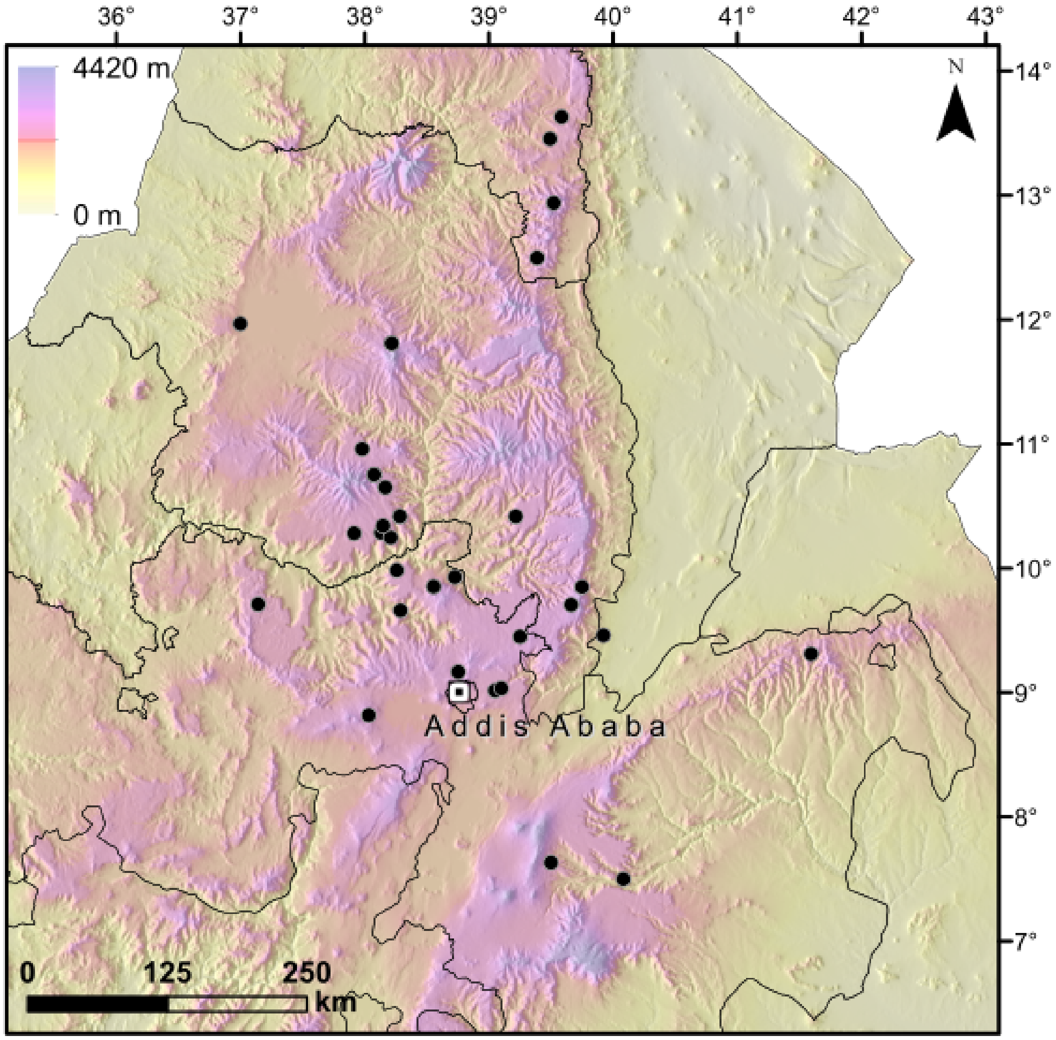

Supplementary Figure 15. Location of origin of the top 41 durum wheat (*Triticum durum* Desf.) genotypes selected for the decentralized trials.

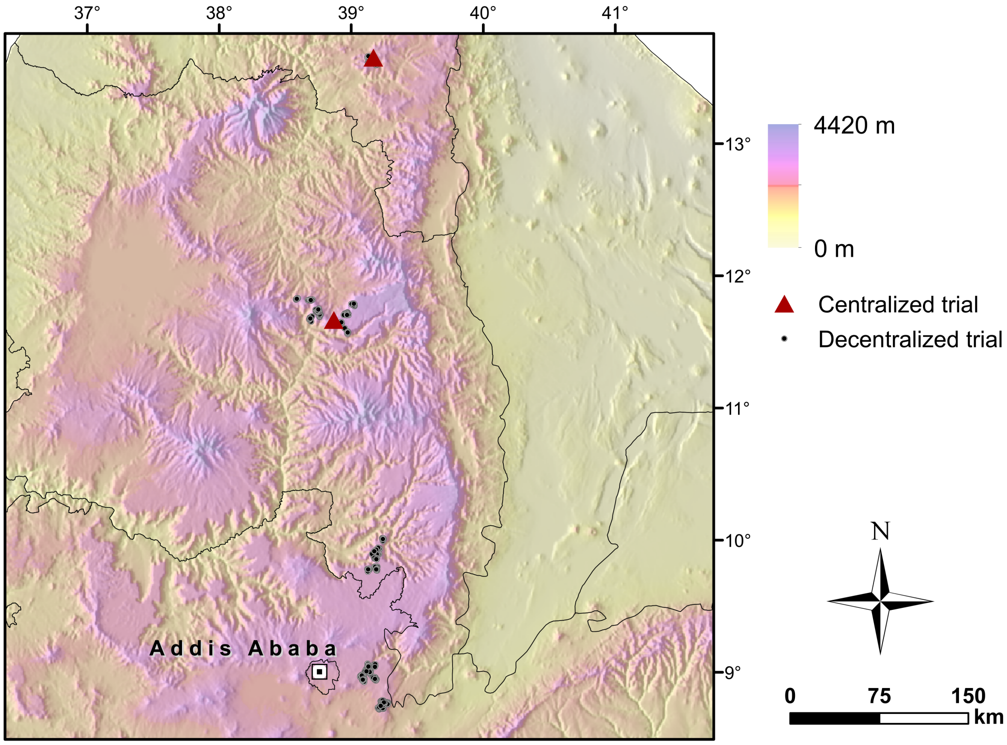

Supplementary Figure 16. Location of decentralized farmers' plots (black dots) and centralized trials (red triangles) across Ethiopia.
