# Supplementary material for: Data-driven decentralized breeding increases prediction accuracy in a challenging crop production environment
Source: Commun Biol. 2021 Aug 19;4:944. doi: 10.1038/s42003-021-02463-w (PMC8376984; doi:10.1038/s42003-021-02463-w)
Supplement: Supplementary file 3 — Description of Additional Supplementary Files [file 42003_2021_2463_MOESM3_ESM.pdf]

Description of Additional Supplementary Files

File name: Supplementary Data 1-4

Description:

Supplementary Data 1: Broad sense heritability (H2) and narrow sense heritability (h 2) of phenotypic traits measured in centralized stations. For each trait, the table reports heritability values for different location and year combinations. DB, days to booting; DF, days to flowering; DM, days to maturity; PH, plant height; NET, number of effective tillers; SPL, spike length; SPS, seeds per spike; BM, biomass; GY, grain yield; TGW, thousands grain weight.

Supplementary Data 2: Broad sense heritability (H2) and narrow sense heritability (h 2) of overall appreciation (OA) provided by farmers in centralized stations. The table reports heritability values for different location and gender combinations.

Supplementary Data 3: Prediction accuracy of the benchmark for GY and OA in different prediction scenarios. For each scenario, the table report a brief description. Training set and validation set are reported with the corresponding number of genotypes in brackets. For both training and validation, total number of data points is derived from number of genotypes, replicas, years, locations. Accuracy and standard deviation of each scenario are provided.

Supplementary Data 4: Mean and standard deviation of the prediction accuracy (Kendall τ) of 3D-breeding in 100 simulations run for each of five reduced scenarios.
