# Supplementary material for: Data-driven decentralized breeding increases prediction accuracy in a challenging crop production environment
Source: Commun Biol. 2021 Aug 19;4:944. doi: 10.1038/s42003-021-02463-w (PMC8376984; doi:10.1038/s42003-021-02463-w)
Supplement: Supplementary file 5 — Reporting Summary [file 42003_2021_2463_MOESM5_ESM.pdf]

nature research

Corresponding author(s): Matteo Dell'Acqua

Last updated by author(s): 25/06/2021

Reporting Summary

Nature Research wishes to improve the reproducibility of the work that we publish. This form provides structure for consistency and transparency in reporting. For further information on Nature Research policies, see our Editorial Policies and the Editorial Policy Checklist.

Statistics

For all statistical analyses, confirm that the following items are present in the figure legend, table legend, main text, or Methods section.

n/a	Confirmed	
☐	☒	The exact sample size (n) for each experimental group/condition, given as a discrete number and unit of measurement
☐	☒	A statement on whether measurements were taken from distinct samples or whether the same sample was measured repeatedly
☐	☒	The statistical test(s) used AND whether they are one- or two-sided<br>*Only common tests should be described solely by name; describe more complex techniques in the Methods section.*
☐	☒	A description of all covariates tested
☐	☒	A description of any assumptions or corrections, such as tests of normality and adjustment for multiple comparisons
☐	☒	A full description of the statistical parameters including central tendency (e.g. means) or other basic estimates (e.g. regression coefficient) AND variation (e.g. standard deviation) or associated estimates of uncertainty (e.g. confidence intervals)
☒	☐	For null hypothesis testing, the test statistic (e.g. F, t, r) with confidence intervals, effect sizes, degrees of freedom and P value noted<br>*Give P values as exact values whenever suitable.*
☒	☐	For Bayesian analysis, information on the choice of priors and Markov chain Monte Carlo settings
☒	☐	For hierarchical and complex designs, identification of the appropriate level for tests and full reporting of outcomes
☒	☐	Estimates of effect sizes (e.g. Cohen's d, Pearson's r), indicating how they were calculated

Our web collection on statistics for biologists contains articles on many of the points above.

Software and code

Policy information about availability of computer code

Data collection

All computer code used to retrieve and analyze data is available through Dataverse (static) and GitHub (dynamic)

Genomic DNA was extracted from fresh leaves pooled from five seedlings for each of the accessions with the GenEluteTM Plant Genomic DNA Miniprep Kit (Sigma-Aldrich, St Louis, USA) following manufacturer's instructions in the Molecular and Biotechnology Laboratory at Mekelle University, Ethiopia. Genomic DNA was checked for quantity and quality by electrophoresis on 1% agarose gel and NanodropTM 2000 (Thermo Fisher Scientific Inc., Waltham, USA). Genotyping was performed on the Infinium 90k wheat chip at TraitGenetics GmbH (Gatersleben, Germany). Single nucleotide polymorphisms (SNPs) were called using the tetraploid wheat pipeline in GenomeStudio V11 (Illumina, Inc., San Diego, CA, USA). SNP calls were cleaned for quality by filtering positions and samples with failure rate above 80% and heterozygosity above 50%. A principal components analysis (PCA) was used to summarize the genetic diversity among samples.

Centralised trials were performed in 2012 and 2013 in the districts of Geregera (Amhara) and Hagreselam (Tigray). In 2012, thirty experienced smallholder farmers growing durum wheat (15 men and 15 women) were invited to participate in the trial evaluations at the station plots, held concurrently after flowering stage. The farmers had no previous knowledge of the genotypes included in this study to prevent bias in the evaluations. The participants provided appraisal with Likert scales (1 to 5 worse to best) given to genotypes for overall appreciation (OA). Research technicians measured grain yield (GY) as grams of grain produced per plot.

A total of 1,165 decentralised plots were performed between 2013 and 2015 during three cropping seasons across the regions of Amhara (471), Oromia (399) and Tigray (295) using a subset of the 41 best genotypes identified through farmer evaluation in centralised trials. Season 1 (2013) comprised 179 fields, Season 2 (2014) comprised 651 fields, and Season 3 (2015) comprised 335 field.

Environmental data was collected from the NASA Langley Research Center Atmospheric Science Data Center Surface meteorological and Solar Energy (SSE) web portal supported by the NASA LaRC POWER Project (https://power.larc.nasa.gov/). We extracted fourteen agroclimatic indices using the R package 'climatrends' using the geographic coordinates from farmer-plots and the planting dates.

Data analysis	All computer code used to retrieve and analyze data is available through Dataverse (static) and GitHub (dynamic)<br><br>All analyses were done using the R software. Grain yield and Overall appreciation measured in centralised trials were used to derive best linear unbiased prediction (BLUP) values using the R package ASReml. The benchmark representing a centralised breeding system was conducted using genomic selection models and marker-based genetic relationship matrices computed on BLUP data with the package rrBLUP. To measure accuracy of genomic selection predictions, we calculated the Kendall's tau coefficient (τ), a measure of similarity of rankings, between predicted values and observed values. The 3D-breeding scenario was developed using the data generated by the citizen science decentralised trials using 'PlackettLuce', an implementation of Plackett-Luce model in R. The Plackett-Luce model estimates for each genotype the probability that it wins, against all other genotypes in the set. To take into account explanatory variables, we created Plackett-Luce Trees (PLT) through model-based recursive partitioning. For model-based recursive partitioning with the PLT we used the variables described in the previous section. The PLT models had a cut-off value of α=0.01 and a minimal group size of 20 percent of the total dataset partitioning selection. We used the PLT models with 100-fold cross-validation in an extension of 3D-breeding, which uses an additive matrix derived from the genotypes' SNP values as a prior for the environmental model associated with the agroclimatic indices described in the later section.

For manuscripts utilizing custom algorithms or software that are central to the research but not yet described in published literature, software must be made available to editors and reviewers. We strongly encourage code deposition in a community repository (e.g. GitHub). See the Nature Research guidelines for submitting code & software for further information.

Data

Policy information about availability of data

All manuscripts must include a data availability statement. This statement should provide the following information, where applicable:

- Accession codes, unique identifiers, or web links for publicly available datasets
- A list of figures that have associated raw data
- A description of any restrictions on data availability

Full data and code is available through Dataverse <https://doi:10.7910/DVN/OEZGVP>. The full project replication workflow is available through GitHub <https://github.com/agrobioinfoservices/tricot-genomic>

Field-specific reporting

Please select the one below that is the best fit for your research. If you are not sure, read the appropriate sections before making your selection.

- [x] Life sciences
- [ ] Behavioural & social sciences
- [ ] Ecological, evolutionary & environmental sciences

For a reference copy of the document with all sections, see nature.com/documents/nr-reporting-summary-flat.pdf

Life sciences study design

All studies must disclose on these points even when the disclosure is negative.

Sample size	Sample size was determined following standard procedures in quantitative genetics. Our study is in line with recent wheat literature both in terms of number of genotypes and number of markers (e.g. <https://www.g3journal.org/content/ggg/9/1/125.full.pdf>). Number of locations/seasons tested in the centralized approach are in line with variety evaluation trials and quantitative genetics studies in wheat (e.g. <https://link.springer.com/article/10.1007/s11032-016-0508-5Z>). Sample size in the crowdsourcing approach exceeds most citizen science approaches used in crop science.
Data exclusions	Only data from genotypes tested in at least two seasons were retained for the analyses.
Replication	Attempts to replicate the analyses were successful.
Randomization	We used a randomised controlled trial design, under the triadic comparison of technologies (tricot) method. Which assigns from a larger pool of items (genotypes) a blind and randomised set of three items as incomplete blocks. The design strives for approximate A optimality, this means that it is robust to missing observations. It also strives for balance for positions of each option. Options are equally divided between first, second, third, etc. position. The strategy is to create a "pool" of combinations that does not repeat combinations and is A-optimal. Then this pool is ordered to make subsets of consecutive combinations also relatively balanced and A-optimal.
Blinding	In both centralised and decentralised trails, farmers had no previous knowledge of the genotypes included in this study to prevent bias in the evaluations. In the decentralised trials, blind sets of three local genotypes plus an improved variety were allocated randomly to farmers as described in the later section. Genotypes were labeled with letters (a, b, c, d).

Reporting for specific materials, systems and methods

We require information from authors about some types of materials, experimental systems and methods used in many studies. Here, indicate whether each material, system or method listed is relevant to your study. If you are not sure if a list item applies to your research, read the appropriate section before selecting a response.

Materials & experimental systems

n/a	Involved in the study
☒	☐ Antibodies
☒	☐ Eukaryotic cell lines
☒	☐ Palaeontology and archaeology
☒	☐ Animals and other organisms
☒	☐ Human research participants
☒	☐ Clinical data
☒	☐ Dual use research of concern

Methods

n/a	Involved in the study
☒	☐ ChIP-seq
☒	☐ Flow cytometry
☒	☐ MRI-based neuroimaging
